# 

**Published:** 1904-03-05

**Authors:** 


### Publications.

Just Published. 8vo. with Photographic reproductions, 3/- net.

**THE PHYSIOGNOMY OF MENTAL DISEASES AND DEGENERACY.** By JAMES SHAW, M.D., formerly Med. Supt. and Co-Licensee Haydock Lodge Asylum. Reprinted, with alterations and additions, from various articles in the "Medical Annual."

Just Published. Crown 8vo. 1/6 net.

**A POCKET BOOK OF CLINICAL METHODS.** By CHAS. H. MELLAND, M.D.Lond., M.R.C.P., Phys. Ancoats Hosp.

THIRD EDITION. 12th Thousand. Small 8vo. Illustrated with 200 Original Drawings, 2/6. Lantern Slides of the Illustrations, 1/- and 1/6 each.

**"FIRST AID" TO THE INJURED AND SICK:** An Advanced Ambulance Handbook. By F. J. WARWICK, B.A., M.B., and A. C. TUNSTALL, M.D., F.R.C.S.

THIRD EDITION. 2/- each, or 27/6 the Set of 16 Sheets, or mounted on linen, 45/- With nickel head.

**(Adopted by the War Office, Admiralty, and London School Board.)**

**LARGE SHEET "FIRST AID" DIAGRAMS:** Being Enlargements of the Illustrations in the above work on sheets 26 in. by 40 in., suitable for lectures and classes.

### MEDICAL ACCOUNTS.

Now is the time to arrange for this troublesome business, and

**WRIGHT'S THREE-BOOK SYSTEM,**
**WRIGHT'S SINGLE-BOOK SYSTEM,**
(11th Edition)
**WRIGHT'S OBSTETRIC RECORDS,**
**WRIGHT'S VISITING LISTS, 1904,**
**WRIGHT'S CHARTS of all kinds,**
(Nearly 400,000 of which have been sold)
**WRIGHT'S CHART HOLDERS,**
(In use all over the world)
**WRIGHT'S CERTIFICATE BOOKS,**
**PROFESSIONAL ACCOUNT FORMS,**
**LETTER HEADINGS, and**
**DISPENSING LABELS.**

Are most of them better than the ordinary run of these things, and their use saves time and trouble.

PLEASE SEND FOR FREE SAMPLES.

**Bristol: JOHN WRIGHT & CO.** **London: SIMPKIN & CO., Ltd.**

---

Second Edition. Crown 8vo., price **3/6** net.

With original "Plea—1900—for the State Registration of Nurses."

**MOCK NURSES OF THE LATEST FASHION.**

By FREDERICK J. GANT, F.R.C.S.,
Consulting Surgeon to the Royal Free Hospital.

BAILLIÈRE, TINDALL & COX,
Henrietta Street, Covent Garden, London.

Crown 8vo. Illustrated. Price **2s. 6d.** net.

**ANÆSTHETICS.** By J. BLUMFELD, M.D.Cantab., Senior Anæsthetist to St. George's Hospital; Anæsthetist to the Grosvenor Hospital for Women and Children, London, and to the National Dental and Metropolitan Hospitals.

*Lancet.*—"....In writing this handbook the author has been quite successful." *Australasian Medical Gazette.*—"We can strongly commend this book." *Medical Chronicle.*—"The book is one which we can heartily recommend to senior students and practitioners."

London: BAILLIÈRE, TINDALL & COX, 8 Henrietta St., Covent Garden.

### Publications—*continued.*

FOURTH EDITION. Plates contain 93 Coloured and Stereoscopic Figures, 209 Illustrations in the Text, **17/6** net, including Stereoscope.

By P. WATSON WILLIAMS, M.D.(Lond.),

Physician in Charge of Throat Department, Bristol Royal Infirmary, &c., &c.

**DISEASES OF THE NOSE, PHARYNX, AND LARYNX.**

WITH A SECTION ON THE EXAMINATION OF

**THE EAR.**

**Press Notices.**—"We know of no better practical guide."—*Practitioner.* "Certain to receive the recommendation of all teachers."—*Brit. Med. Journ.* "We can strongly recommend the book."—*Lancet.* "Excellent.... The features of the work—completeness, clear description, and practical utility—are absolutely maintained in the new edition.—(Transn.)." *Internat. Centr. f. Laryngologie.*

Bristol: J. WRIGHT & CO. London: SIMPKIN & CO.

JUST ISSUED. EIGHTH EDITION. Crown 8vo, **10s. 6d.**

**PHARMACY, MATERIA MEDICA AND THERAPEUTICS**

Is now rewritten and printed in large type, and is made a companion to the following volume. It contains an account of the action **of every drug in the B.P.**, as well as a section upon all the newest drugs, with an Introduction to the Art of Dispensing. With woodcuts, prescriptions, &c.

Also FOURTH EDITION. 1055 pages, 8vo. **16s.**

**DICTIONARY OF TREATMENT.**

Containing a Revised and up-to-date Article upon every Diseased Condition, whether Medical, Surgical, Gynæcological, or Ophthalmological, as well as Articles upon Poisoning, Drowning, and the various Surgical Emergencies and Accidents, arranged for Rapid Reference.

By W. WHITLA, M.A., M.D., Professor of Materia Medica, Queen's College, Belfast; Senior Physician to the Royal Victoria Hospital, &c.

The Publisher will supply both volumes, carriage paid, for **21/-**

London: HENRY RENSHAW, 356 Strand.

**Hoblyn's Dictionary of TERMS USED IN MEDICINE and the Collateral Sciences.**

13th Edition, Revised by J. A. P. PRICE. **10/6.**

"It has a rare merit of supplying in almost every case what you have a right to expect in consulting it."—*Glasgow Medical Journal.*

"As a handy reference volume for the physician, surgeon and pharmacist it will prove invaluable."—*Pharmaceutical Journal.*

**THE APOTHECARIES' HALL MANUAL FOR Students preparing for the Dispenser's Certificate. By M. THOMSON, 2 - net.**

WHITTAKER & CO., White Hart St., Paternoster Sq. London, E C.

NOTICE TO CORRESPONDENTS.
All MSS., Letters, Books for Review, and other matters intended for the Editor should be addressed The Editor, "The Hospital," The Hospital Buildings, 28 & 29 Southampton Street, Strand, W.C.
The Editor cannot undertake to return rejected MS., even when accompanied by stamped directed envelope.

**Notice to Advertisers and Subscribers.**
All Advertisements, Orders for Copies of The Hospital, and Business Communications should be addressed to **THE MANAGER (Not to the Editor),** The Hospital, 28 & 29 Southampton Street, Strand, London, W.C.
The Cost of Subscription, post free, is as follows:—Per week, 3½d.; per quarter, 3s. 9d.; per half-year, 7s. 6d.; per annum, 15s. Foreign and Colonial:—Per annum, 19s. 6d., payable, in all cases, in advance.

**NOTICE.—Vol. XXXIV. of "The Hospital" (April 1903 to September 1903), in handsome cloth binding, is now on sale at the office of this Journal, price 10s. 6d. Cases for binding the Numbers contained in this Volume 2s. Index to Vol. XXXIV., 2½d., post free.**
**The Monthly part for March is now ready. Price 1s., by post, 1s. 3d.**

### Important

**To Midwives,**

**Nurses and**

**Probationers.**

IN THE PRESS. READY SHORTLY.

### A COMPLETE HANDBOOK
FOR
### MIDWIVES.

By J. K. WATSON, M.D. M.B., C.M.,

Author of "A Handbook for Nurses," "Examination of the Urine," &c., &c.

**6/-** net
**6/4**
post free.

This handbook has been designed to meet the requirements of the Central Midwives Board, and is an up-to-date text-book of **all** that the midwife requires to know of the subject treated. The work is produced with the object of rendering it unnecessary for midwives, during their preparation for examination, to consult the larger and more expensive works on the subject written specially for medical men.

**The illustrations are very complete, numbering some 130 separate diagrams, most of which have been specially prepared for this book.**

**6/-** net
**6/4**
post free.

Crown 8vo., about 350 pp., bound in cloth boards.

*N.B.*—As the demand for this work is expected to be very great, Nurses are advised to order the book without delay, as the first edition will in all probability soon be exhausted.

*Of all Booksellers, or of*

THE SCIENTIFIC PRESS, LTD., 28 & 29 Southampton Street, Strand, London, W.C.

### WORKS FOR NURSES.

**LESSONS ON MASSAGE.** By Mrs. MARGARET D. PALMER, Manager of the Massage Department and Instructor of Massage to the Nursing Staff of the London Hospital. Second Edition enlarged and revised; with 118 Illustrations. Price 7/6 net.

**A MANUAL FOR STUDENTS OF MASSAGE.** By Miss M. A. ELLISON, L.O.S. Second Edition, edited by Miss G. MANLEY, pp. xii + 126. With 50 original Illustrations. Price 3/6 net.

**A MANUAL FOR HOME NURSING.** By BERNARD MYERS, M.D., M.R.C.S., Surgeon and Lecturer to St. John's Ambulance Association. 2/6 net.

**NOTES ON HOME NURSING.** By Miss D. M. GOLDIE, Lecturer on Nursing, Hygiene, &c., to the County Councils of London, Surrey, &c. 1/6 net.

**NUTRITION OF THE INFANT.** By RALPH VINCENT, M.D., M.R.C.P., Physician to the Infants' Hospital.

**THE FEEDING AND HYGIENE OF INFANTS AND CHILDREN.** By JOHN McCAW, M.D., M.R.C.P., Physician to the Belfast Hospital for Children. *Just Published.* Price 2/6.

**MIDWIFERY FOR MIDWIVES.** Designed to cover the requirements of the Examining Board authorised by the Midwives Act. With Appendix on Examinations. By W. DENISON WIGGINS, M.R.C.S., L.R.C.P., &c. *Just out.* 3/6 net.

**DISEASES OF THE HAIR, INCLUDING GREYNESS, BALDNESS, &c., AND THEIR TREATMENT.** By DAVID WALSH, M.D., Western Hospital for Diseases of the Skin. Price 2/6 net.

**MENSTRUATION AND ITS DISORDERS.** By ARTHUR E. GILES, M.D., B.Sc., F.R.C.S., Surgeon Chelsea Hospital for Women. Price 2/6 net.

**MOVABLE ATLAS OF THE ANATOMY AND PHYSIOLOGY OF THE FEMALE GENERATIVE ORGANS AND OF PREGNANCY.** Second Edition. With Text. By ARTHUR E. GILES, M.D., F.R.C.S. Price 3/- net.

**MOVABLE ATLAS OF THE ANATOMY AND PHYSIOLOGY OF THE CHILD.** With Explanatory Text. By D'ARCY POWER, M.A.Oxon, M.B., F.R.C.S. Price 3/- net.

London: BAILLIÈRE, TINDALL & COX, 8 Henrietta Street, Covent Garden.

